# Perspective: Integration to Implementation (I-to-I) and the Micronutrient Forum—Addressing the Safety and Effectiveness of Vitamin A Supplementation

**DOI:** 10.1093/advances/nmz100

**Published:** 2019-09-30

**Authors:** Daniel J Raiten, Ian Darnton-Hill, Sherry A Tanumihardjo, Parminder S Suchdev, Emorn Udomkesmalee, Carolina Martinez, Dora Inés Mazariegos, Musonda Mofu, Klaus Kraemer, Homero Martinez

**Affiliations:** 1 Eunice Kennedy Shriver National Institute of Child Health and Human Development, National Institutes of Health, Bethesda, MD, USA; 2 The Boden Collaboration for Obesity, Nutrition, Exercise, and Eating Disorders, Faculty of Medicine and Health, University of Sydney, Sydney, New South Wales, Australia; 3 The Gerald J and Dorothy R Friedman School of Nutrition Science and Policy, Tufts University, Medford, MA, USA; 4 Department of Nutritional Sciences, University of Wisconsin-Madison, Madison, WI, USA; 5 Department of Pediatrics and Emory Global Health Institute, Emory University, Atlanta, GA, USA; 6 Department of Human Nutrition, Institute of Nutrition, Mahidol University, Salaya, Nakhon Pathom, Thailand; 7 Instituto de Nutrición de Centro América y Panamá (INCAP), Guatemala City, Guatemala; 8 National Food and Nutrition Commission, Lusaka, Zambia; 9 Sight and Life, Basel, Switzerland; 10 Johns Hopkins Bloomberg School of Public Health, Baltimore, MD, USA; 11 Nutrition International, Ottawa, Ontario, Canada; 12 Hospital Infantil de México Federico Gomez, Mexico City, Mexico

**Keywords:** integration to effective implementation (I-to-I), vitamin A supplementation, safety, effectiveness, transition, policies and programs, health promotion and disease prevention, nutrition-sensitive, nutrition-specific

## Abstract

An ongoing challenge to our ability to address the role of food and nutrition in health promotion and disease prevention is how to design and implement context-specific interventions and guidance that are safe, efficacious, and avoid unintended consequences. The integration to effective implementation (I-to-I) concept is intended to address the complexities of the global health context through engagement of the continuum of stakeholders involved in the generation, translation, and implementation of evidence to public health guidance/programs. The I-to-I approach was developed under the auspices of the Micronutrient Forum and has been previously applied to the question of safety and effectiveness of interventions to prevent and treat nutritional iron deficiency. The present article applies the I-to-I approach to questions regarding the safety and utility of large-dose vitamin A supplementation programs, and presents the authors’ perspective on key aspects of the topic, including coverage of the basic and applied biology of vitamin A nutrition and assessment, clinical implications, and an overview of the extant data with regard to both the justification for and utility of available intervention strategies. The article includes some practical considerations based on specific country experiences regarding the challenges of implementing vitamin A–related programs. This is followed by an overview of some challenges associated with engagement of the enabling communities that play a critical role in the implementation of these types of public health interventions. The article concludes with suggestions for potential approaches to move this important agenda forward.

## Introduction

**FIGURE 1:**
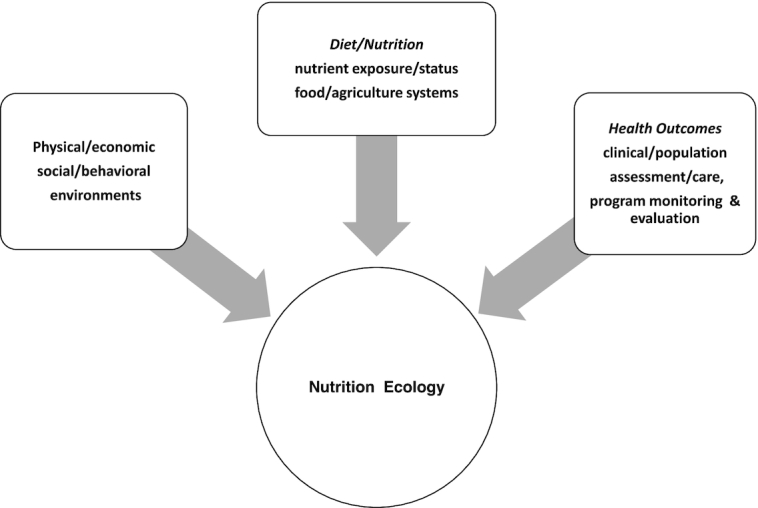
Nutrition ecology: program implementation and service delivery need to be continually reconciled with the new evidence generated throughout the continuum of effort and the biological and resource context “on the ground.”

**FIGURE 2:**
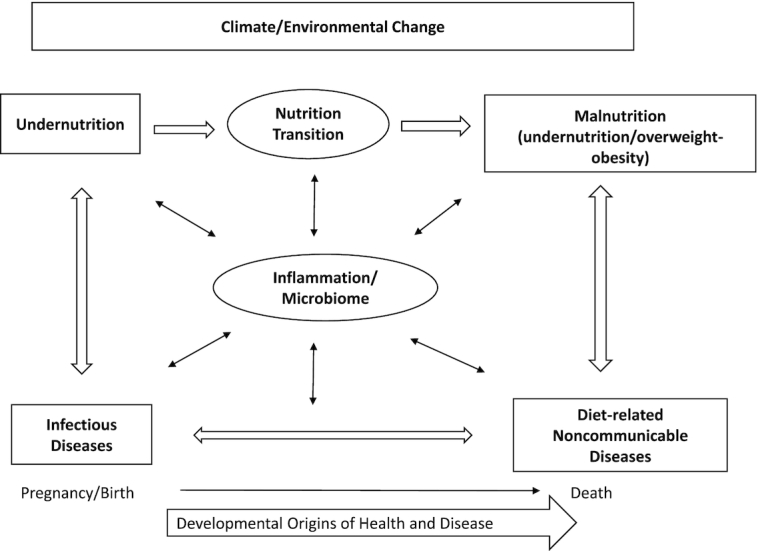
The global nutrition and health context. Adapted from reference 1 with permission.

The “integration to effective implementation” (I-to-I) concept has been previously introduced by the Micronutrient Forum (1). Briefly stated, the I-to-I approach is an effort to promote global health by providing a comprehensive framework to address: *1*) the range of components of the “nutritional ecology” (**Figure 1**), and *2*) the need to engage the multiple stakeholders involved in the continuum of effort toward the development and implementation of evidence-informed programs, policies, and standards of care. The application of the I-to-I approach would also help address concerns that arise about the safety and/or efficacy of a particular public health intervention resulting from new knowledge, accumulated experience, or changing/evolving conditions in the ecosystem.

With regard to nutrition-specific interventions (2), it is essential to recognize what triggers are associated with these interventions, such as the overall status of a given nutrient in the population, including micronutrient supplementation to address inadequate dietary intake, the biological variables affected by specific deficiencies, and physiological interactions among numerous processes. These processes include ingestion, digestion, metabolism, utilization, and integration into key biological systems resulting in functional outcomes, for example, growth, immunocompetence, response to therapeutics, development, and neurological function. Nutritional status and the processes by which it is achieved have a reciprocal relation with the health of the patient and with the target populations served by public health interventions and standards of care. The complexity of the intersection of nutrition and the global health context is outlined in **Figure 2**.

Not only is the interaction between infectious and noncommunicable diseases (NCDs) well recognized (3, 4), but also the emerging understanding of the roles of early life nutrition and of the microbiome in mediating this interaction is now critical for better understanding the complexity of global health. An additional consideration is the growing appreciation of the inflammatory process as both a mediator of nutrition, and at least of equal importance, its impact on the interpretation of many of the biomarkers used to assess nutritional status (4, 5).

Considering the complexity of this nutritional ecology, it becomes clear that the approach to addressing malnutrition (i.e., undernutrition/overweight-obesity, representing a double burden of malnutrition) is more than a matter of having “too much” or “too little” of 1 or several macronutrients/micronutrients (micronutrient deficiencies adding a further triple burden of malnutrition) or energy/nutrient density in the diet. As we learn more about these interrelations, we will need to improve our processes for translating new data/knowledge in a timely fashion to develop safe (do no harm) and efficacious interventions that achieve positive health outcomes, are culturally, economically, and environmentally sustainable, and avoid unintended consequences. The I-to-I approach is intended to facilitate the dialogue amongst the relevant stakeholders to achieve these goals.

An evolving view of this continuum includes a number of components involved in efforts to develop and implement public health interventions. This view also recognizes that the process is not linear but circular, involving a continued process of generating and interpreting new data inputs and outcomes. These elements are listed in **Table 1**.

**TABLE 1:** Components of the nutrition enterprise covered by the I-to-I approach^1^

*Basic biomedical/clinical/plant/environmental/animal science research* To understand the nature and mechanism of problems related to the many aspects of human growth and development
*Knowledge translation* Best practices in clinical assessment and surveillance to identify problems in individuals and populations
*Interventions* Sustainable, culturally relevant, and biologically based nutrition-specific and nutrition-sensitive interventions and programs
*Implementation (both rolling-out and scaling-up)* Must involve a range of stakeholders at local/community, national, and global levels
*Monitoring and evaluation* of programs/policies Timely and responsive change as needed. Data collection to give feedback at critical points throughout a continuum that includes research, translation, intervention development/revision, etc.
*Data inputs/outputs* Creation of a critical interconnecting and continuous loop to enable responsiveness to changes anywhere along the chain, allowing for the correcting/avoiding of unintended consequences

1 I-to-I, integration to implementation.

### I-to-I case study II: vitamin A supplementation programs

In the first application of the “I-to-I” approach, the concerns about the safety and efficacy of interventions to prevent and treat iron deficiency were addressed (1). These concerns arose as a result of documented adverse outcomes in the context of infections, such as malaria, the challenges of assessing iron status in the presence of inflammation, and lack of clarity with regard to the safety and efficacy of the various available options for preventing and treating iron deficiency. The implications of these concerns were addressed in terms of not only new scientific knowledge, but also the impact on those involved in trying to address this major health concern from local/country to global levels.

The present article summarizes the second effort to apply the I-to-I model to another high priority public health issue, that of the safety and efficacy of interventions to prevent vitamin A deficiency (VAD). This article is based on the presentations and subsequent deliberations of the coauthors initiated at a symposium sponsored by the Micronutrient Forum and conducted at the 2017 International Congress of Nutrition meeting in Buenos Aires. The article is organized into several components covering the core areas of: vitamin A biology, clinical considerations, current program/intervention options, and issues pertaining to the engagement of the enabling communities needed to implement and support such programs. The article also includes perspectives provided by individual country experiences with specific regard to vitamin A.

## Current Status of Knowledge

### Estimates of VAD

Current estimates of the prevalence of VAD at the country level are limited or missing in most low- and middle-income countries (LMICs) (6). However, data from the WHO indicate that the global prevalence of night blindness (a biological indicator associated with clinical VAD) includes 5.17 million preschool-age children and 9.75 million pregnant women. Based on a serum retinol concentration <0.7 μmol/L, it is estimated that 190 million preschool-age children could be affected, as well as 19.1 million pregnant women (7).

The root cause of VAD is insufficient intake, but vitamin A status can also be impacted by acute and chronic inflammation associated most prominently with infectious disease; status can be further compromised by increased utilization and excretion. Consequently, complementary efforts to improve vitamin A status should include actions directed to the prevention and control of infectious diseases. In a virtuous biological cycle, improving vitamin A status will also reduce the number of deaths from infectious diseases in children in LMICs (8–10).

With specific regard to intake, the safest and most sustainable intervention for improving vitamin A status is improved dietary diversity and related food-based approaches to provide better access to sources of vitamin A. Unfortunately, there are often challenges to improving vitamin A status through improved food/nutrient security and dietary diversity, thereby necessitating other intervention options, most prominently fortification of staple foods. For example, fortification of sugar, oils and margarine, and wheat flour has been successfully implemented in many countries that have government-regulated programs (11–13). Another variation of this approach is the use of biofortified foods like orange-fleshed sweet potatoes or maize providing provitamin A carotenoids. Because biofortification involves the provision of provitamin A to the diet, consideration needs to be given to associated changes related to bioavailability and bioconversion (14–16).

In countries or regions where improved dietary access or fortification are either not viable/effective options or do not guarantee full coverage for at-risk populations, vitamin A supplementation (VAS) has proven to be a useful option. VAS is recommended by the WHO (17) and includes the periodic (semiannual) delivery of 2 doses of 200,000 IU of vitamin A to preschool-age children (1 to <5 y of age) and 1 dose of 100,000 IU of vitamin A to infants 6–11 mo old (18). Global coverage peaked at 76% in 2009, but by 2016 only 64% of children in need received the recommended 2 doses of annual vitamin A—a drop by more than a half in countries with the highest under-5 mortality rates (19).

### The controversy

Numerous options exist to address VAD as a public health issue including food-based interventions (e.g., dietary diversification, fortification, biofortification, or various modes of dietary supplementation). As will be discussed below many of these options have shown efficacy in particular contexts. Nevertheless, on a global scale, 6-monthly supplementation with high-dose vitamin A capsules to young children (usually aged <6 y) has been shown to be efficacious and remains the main approach (20). According to UNICEF, the use of this approach peaked in 2009 at 78% (∼290 million children) in 27 priority countries (19). In 2016, 89% of governments had a policy, strategy, or plan of action on VAS (21). In sub-Saharan Africa only 10 countries (down from 27 in 2009) had active programs. A similar downward trend in coverage has been reported globally, where coverage is now only 64% in priority countries (19). As a public health intervention, questions have been raised about the sustainability of these supplementation programs and whether the decline in use is associated with diminished effectiveness as the health and nutrition profiles of countries change. In addition, reliance on donors and cooperating partners for the financing of supplementation is of further concern. As a consequence, assistance to countries for different complementary approaches, including fortification, dietary diversification, biofortification, and multimicronutrient powders, is now gaining increased attention (22–24). It is against this backdrop that questions have been raised about the evidence used to justify the use of high-dose VAS.

The justification for high-dose VAS is less about serving as a sustainable method to improve vitamin A status and more about reducing mortality due to such infectious diseases as measles and acute diarrhea in vitamin A–deficient young children (25, 26), presumably via still poorly understood interactions with other immunomodulatory interventions, including bacille Calmette–Guérin (BCG) and diphtheria-tetanus-pertussis (DTP) vaccines (27). Recent questions have been raised as a result of 2 core factors: *1*) vitamin A status improves only transitorily (1–3 mo) in children with low dietary intake following high-dose VAS (28), and *2*) evidence suggests that vitamin A might not be as effective as previously thought in reducing child mortality (27, 29, 30). A recent Cochrane review supported the efficacy of VAS (31), but the controversy regarding efficacy persists. In addition, concerns have been raised about the sustainability of VAS programs. These questions have led some to recommend shifting the current policy supporting VAS toward a range of other interventions, including food-based approaches (e.g., fortification) or regular low-dose supplementation or even total cessation of current high-dose VAS (32, 33). Other bodies have argued strongly against the last, until affected countries show impact on under-5 mortality rates (8, 34).

Currently, >80 countries worldwide have universal VAS programs that deliver high-dose vitamin A to children aged 6–59 mo on a biannual basis. The Global Alliance for Vitamin A (GAVA) recommends that countries should only consider scaling down this intervention when it is no longer considered a public health problem, that is, when there is evidence from ≥2 nationally representative cross-sectional surveys showing that VAD prevalence (serum retinol <0.7 μmol/L) is <5% (35). More recent (2019) guidance suggests that scaling back VAS can occur when VAD prevalence is <10% and dietary intake data indicate adequate vitamin A intake (36).

The objective of this article is to provide a perspective on the need for a comprehensive approach to address vitamin A insufficiency, considering basic biology, clinical/population assessment, and different interventions, and how these can be translated to appropriate programs and policies using the I-to-I approach.

The following sections summarize:

- Basic vitamin A biology and assessment
- Clinical perspectives
- Relative strengths and weaknesses of vitamin A interventions
- Country perspectives: implications of the current concern/debate on national efforts to prevent VAD
- Context and perspectives from enabling agencies

### Basics in vitamin A biology and assessment

**FIGURE 3:**
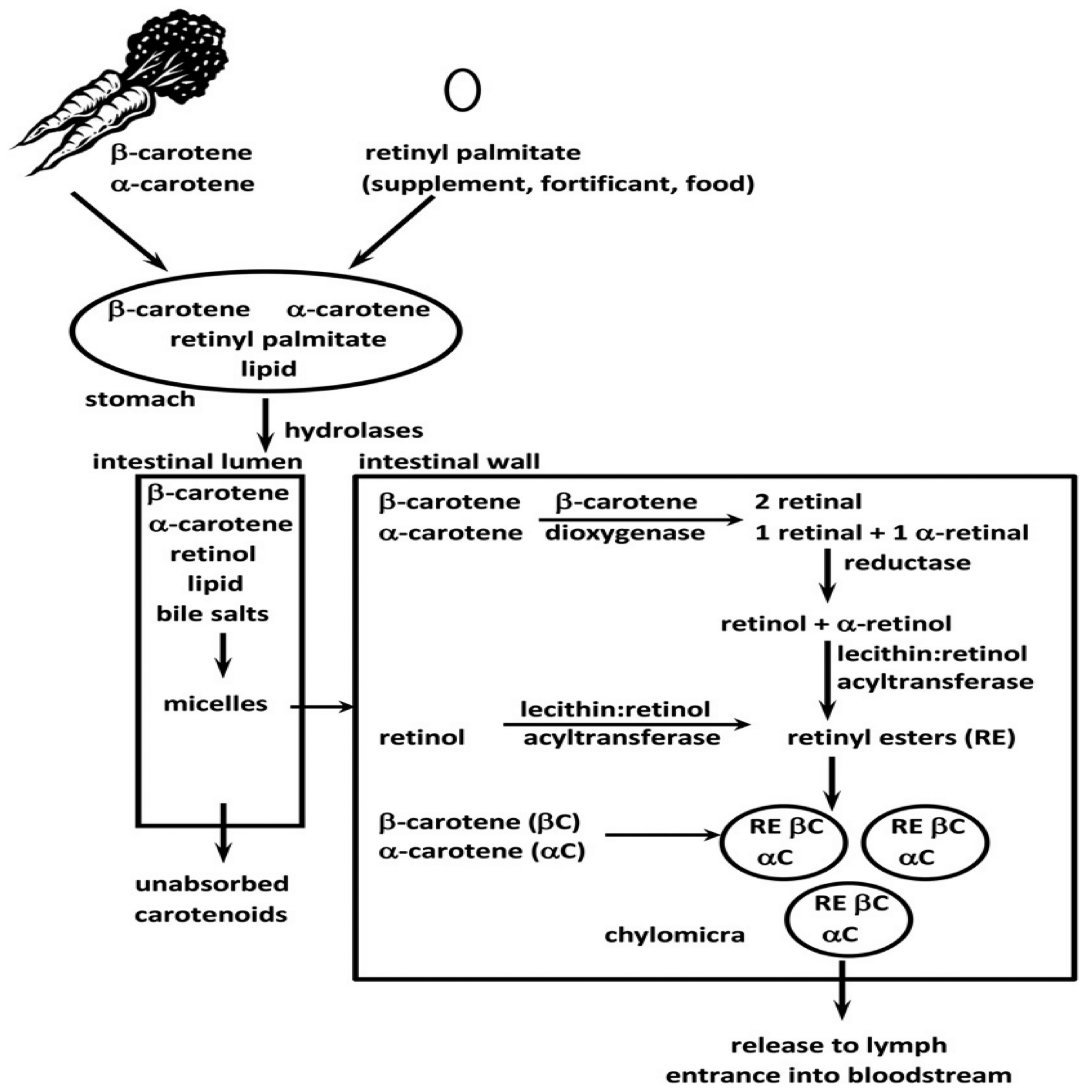
Major forms of vitamin A, provitamin A carotenoids, and vitamin A metabolism. αC, α-carotene; βC, β-carotene. Reproduced from reference 37 with permission.

An overview of the major forms of vitamin A, provitamin A carotenoids, and vitamin A metabolism is depicted in **Figure 3** (37). The primary form found in vitamin A supplements is retinyl palmitate, which, when given at high doses, results in increased fecal excretion to avoid “overwhelming the system” (38). The most common provitamin A carotenoids in the human diet include α- and β-carotene and β-cryptoxanthin. Carotenoids from food are not as bioavailable as those from supplements, and therefore the Institute of Medicine recommends that a bioefficacy factor of 12 μg β-carotene equivalents from a mixed diet be equivalent to 1 μg of retinol in dietary calculations of generally healthy individuals (39).

Vitamin A in the human body is found predominantly in the liver. A variety of biomarkers exist to assess vitamin A status (37). The 2 most common biomarkers available for population surveys include serum retinol concentrations and the modified relative dose response (MRDR) test. Both require only a single blood sample, which is desirable for field surveys. Furthermore, analysis only requires HPLC, which is usually available in countries that choose to use these biochemical markers. Each measure has different merits compared with other available biomarkers for vitamin A concentrations in the body:

- Serum retinol, the most commonly used biomarker, is a static index of vitamin A that is homeostatically controlled and does not respond substantially to vitamin A supplementation unless the subject is severely vitamin A deficient.
- The MRDR test reflects liver stores and responds to low storage concentrations of retinol in the liver. The MRDR value is almost invariably negative when liver reserves are high (38).

The MRDR test gives more discrimination than serum retinol concentrations alone, showing that it has greater potential to yield more statistical power on fewer subjects and a better definition of the actual vitamin A status of the population (38). Demonstrating this, in 2 studies, one in Zambia (40) and another in Indonesia (41), no change in serum retinol was reported in response to vitamin A interventions but MRDR values, reflective of liver stores, did respond in both trials. Of additional interest was that in the Zambian cohort, both groups of children (traditional unfortified white maize compared with carotenoid-enhanced maize) lost liver stores over the course of the intervention, demonstrated by an increase in MRDR values. In the study in Indonesia, the MRDR test could distinguish between children who had received a high-dose supplement and those who had not, even though serum retinol concentrations were not different between the groups (42).

The most commonly accepted tenets of vitamin A homeostasis are that: *1*) total body vitamin A stores regulate vitamin A homeostasis (43); *2*) vitamin A status indirectly regulates bioconversion of provitamin A carotenoids to retinol (44–46); and *3*) bioconversion in the intestine is regulated through diet-responsive regulatory mechanisms (47). However, it is important to note that the role and extent to which liver stores regulate provitamin A carotenoid absorption and/or bioconversion have also not been thoroughly determined in humans (47). Of interest is a report in which Zambian children with hypervitaminosis A, diagnosed using stable retinol isotope dilution, had high serum carotenoid concentrations (48). The children in this cohort also demonstrated hypercarotenodermia during the mango season, presumably associated with high carotenoid intake (49). The authors surmised that these results indicate that even in the face of high liver stores and presumptive hypervitaminosis A, carotenoid absorption still occurred and these carotenoids were stored intact in tissues.

The current uncertainty on the appropriate retinyl palmitate supplementation dose is being examined through studies on the basic biology of vitamin A homeostasis after high-dose supplementation using animal models. Animal studies with the lactating sow–nursing piglet dyad have revealed interesting results concerning response to different levels of supplementation. Offspring of sows that had received the human equivalent of either 200,000 IU or 400,000 IU had the same liver retinol reserves at time of kill (50). In piglets receiving graded doses of retinyl ester, liver vitamin A reserves were only 5% higher in piglets receiving 100,000 IU than in those receiving 50,000 IU. Liver retinol responded dose dependently, but serum retinol concentrations did not differ (51). MRDR values were much higher in the piglets from sows that had been on a vitamin A-depleted diet for longer. In a comprehensive study that evaluated newborn supplementation in a piglet model, 0, 25,000, 50,000, and 200,000 IU of retinyl palmitate were administered at birth. Liver retinol reserves did not differ between 25,000 and 50,000 IU and 50,000 and 200,000 IU at most time points, with much lower retention of the 200,000 IU dose (21). Another finding revealed that growth of piglets in the newborn study was interrupted when they received the vitamin A supplements compared with the placebo group. In a study in vervet monkeys, weight was also negatively impacted with higher vitamin A status (52). The studies in both pigs and monkeys provide evidence not only to support the importance of monitoring liver stores (through MRDR rather than only circulating concentrations of retinol) (51) but also of potential adverse effects on growth of high-dose vitamin A (21) or lifetime exposure to vitamin A fortification in their feed (52).

In conclusion, the mantra of “more is better” clearly does not apply to intervention programs designed to improve vitamin A status. The most commonly used biomarker for assessing vitamin A status, serum retinol, does not fully reflect vitamin A biology due to homeostasis, particularly in terms of predicting potential liver stores and toxicity. The MRDR test and other tools can be more effective at predicting unintended consequences of vitamin A interventions. More research is needed to expand our understanding of vitamin A homeostasis, including the regulation of carotenoid absorption and bioconversion to retinol and how the body responds to high liver stores of vitamin A in terms of feedback on catabolic pathways. Vitamin A and its precursors represent a complex biological system that impacts, and is impacted by, other biological systems integral to normal growth and function. In the absence of a fuller understanding of these interrelations, a balanced approach based on available evidence is needed to assess and to inform the process for making decisions about the current tools for preventing and treating VAD in low-resource settings.

### Clinical perspective

Regarding how best to address questions about vitamin A treatment options, the core questions for clinicians are focused on how to assess vitamin A status, particularly in the context of the patient's overall health status, how to interpret those results, and what to do next? The answers are rarely straightforward. These challenges are illustrated by the case of a 2-y-old child being managed as an outpatient for severe acute malnutrition with fortified ready-to-eat therapeutic food (RUTF) who presents to clinic with diarrhea and poor appetite. Two weeks prior to this visit, a measles campaign with high-dose VAS took place in the child's village. Here are 3 options:

- Provide high-dose VAS and admit to malnutrition treatment center?
- Admit for treatment of malnutrition, but do not administer vitamin A, because the child is already receiving the daily recommended intake?
- Provide oral rehydration and zinc and send home?

It can be argued that the child in the case study described should not continue to be managed as an outpatient, because she or he has an acute illness with poor appetite. Only children with severe acute malnutrition who have no apparent problems with appetite and are otherwise clinically well should be treated as outpatients. In the case of the outpatient child, the choice of oral rehydration therapy plus zinc could be inappropriate. As for the use of a high-dose VAS, because the child is already receiving vitamin A via the fortified RUTF being used to treat their severe acute malnutrition, providing a further high-dose VAS might not be the first choice of action, although the provider would want to confirm whether the child actually received the supplement. If intake was impaired due to the illness (diarrhea), then VAS could also be given. It can then be argued that admission for the illness, but no additional VAS, would be the best course of action (53). The bottom line in this decision-making process is that context matters.

An estimated 30% of children are vitamin A deficient globally based on serum retinol, contributing to 2% of childhood deaths (8). In response, WHO currently has 10 guidelines specific to VAS in various target groups, including neonates and young children (18, 54), pregnant and postpartum women (55), and individuals with clinical illness, including severe acute malnutrition, human immuno-deficiency virus, and respiratory infections (56, 57). The goals of VAS are broadly to:

- Decrease night blindness and xerophthalmia.
- Reduce child mortality (by 12–24%, as above) likely by reducing case fatality of measles, diarrhea, and other infections.
- Improve vitamin A nutrition, although as described above, any improvement in status is short-lived, so other ongoing dietary and public health measures are likely to be needed.

VAS of preschool children is one of the most widely used public health interventions, implemented in >80 countries worldwide with an estimated current median coverage of 70% (58). Despite the clinical importance of addressing VAD, there are critical gaps in the population vitamin A status data that are needed to appropriately target interventions. In a recent review of 82 countries with VAS programs, two-thirds had no VAD data, or data that were >10 y old (59). Fifty-one VAS programs were implemented in parallel with ≥1 other vitamin A intervention, and of these, 27 countries either had no VAD data or data collected in 2005 or earlier, as shown in **Table 2**. To inform clinical and public health programs and policy, more accurate estimates of vitamin A status are needed.

**TABLE 2:** Profile of VAS programs^1^

• Of 82 countries implementing VAS, 77 had coverage data
• 54 were running in parallel with ≥1 other vitamin A intervention:
✓ 41 also had mass fortification (vegetable oils, sugar, margarine, wheat flour)
✓ 21 had biofortification (sweet potato, maize); 17 of which also had vitamin A fortification
✓ 21 implemented micronutrient powders; of which 12 also had fortification or biofortification
• Of these 54, 27 had no data or data were older than 10 y
• Vitamin A assessment under micronutrient survey including program coverage should be done every 10 y

1 Data from reference 19 (UNICEF, 2018). VAS, vitamin A supplementation.

However, as noted above, the availability of field-friendly and valid biomarkers for VAD remains a gap (22). An added complexity are the implications of the acute-phase response for interpreting biomarkers of vitamin A status, because both serum retinol and retinol-binding protein (RBP) are negative acute-phase reactants (i.e., concentrations decrease in the presence of inflammation), thereby creating the potential for overestimating VAD if not adjusted for inflammation (22, 50). Data from the Biomarkers Reflecting Inflammation and Nutritional Determinants of Anemia (BRINDA) project attest to the implications of inflammation for vitamin A assessment (60). In an analysis of 20 surveys of preschool children from Asia, sub-Saharan Africa, and North, Central, and South America, both retinol and RBP concentrations were significantly negatively correlated with C-reactive protein and α_1_-acid glycoprotein concentrations, and adjusting for this effect decreased the estimated prevalence of VAD by a median of 13.1 to 16.4 percentage points (for retinol compared with RBP, respectively) (61). Using unadjusted retinol and RBP concentrations, 14 countries had an estimated VAD prevalence of >20%, considered a severe public health problem by the WHO. However, after adjusting for inflammation using the BRINDA approach, the public health significance of VAD dropped from severe to mild in 7 surveys and from severe to moderate in 4 surveys (61). These data demonstrate the importance of accounting for inflammation when assessing the population prevalence of VAD.

Clinical considerations need to be integrated into public health programs tackling VAD. These might include prevention and treatment of general malnutrition, prevention and treatment of infections, and supplementation of individuals with vitamin A when warranted. With regard to the use of VAS, as noted above, context matters and care should be taken to address all contingencies regarding vitamin A status including the potential for toxicity which, although rare, does happen (62–64).

### Relative strengths and weaknesses of vitamin A interventions: evidence base and lessons learned

This section focuses on the application of current knowledge of interventions to address VAD. As noted above, the analyses presented by Mason et al. (32) have stimulated considerable debate about the current justification used in defense of high-dose VAS, that is, that these interventions both improve vitamin A status over time and reduce child mortality. An analysis of the extant data reveals both the slowing trends in reduction of VAD (as assessed with serum retinol concentrations and the WHO cutoff point of 0.7 μmol/L) and a relatively flat trend in child survival provided by VAS programs, as well as a relative disconnect from trends in child mortality. To put this argument in perspective, based on these analyses VAS is not expected to reduce VAD prevalence, yet it nevertheless is promoted as a child survival intervention.

Contrasting with the position of Mason et al., an earlier Cochrane systematic review covering 43 randomized clinical trials involving 215,633 children had shown a 24% reduction in all-cause mortality in children aged <5 y receiving high-dose supplementation using commercially produced vitamin A capsules (65). Caveats raised by Mason et al. (32) regarding total reduction in all-cause mortality include the observation from data analyses of the original Aceh Trial that the most pronounced reductions in mortality were in children 2 mo postdose, with rates stalling afterwards. This might indicate a critical window for the efficacy of VAS compared with other intervention options. It was also suggested that if the data from the Deworming and Enhanced Vitamin A Supplementation Trial (29) were added to the Cochrane data, the effect would be cut in half to a 12% reduction in all-cause mortality. It should be noted that although this is a significant reduction in the currently accepted effect size, a 12% reduction in mortality still represents a significant public health outcome that provides justification for the continuation of WHO guidance with regard to VAS (17).

**FIGURE 4:**
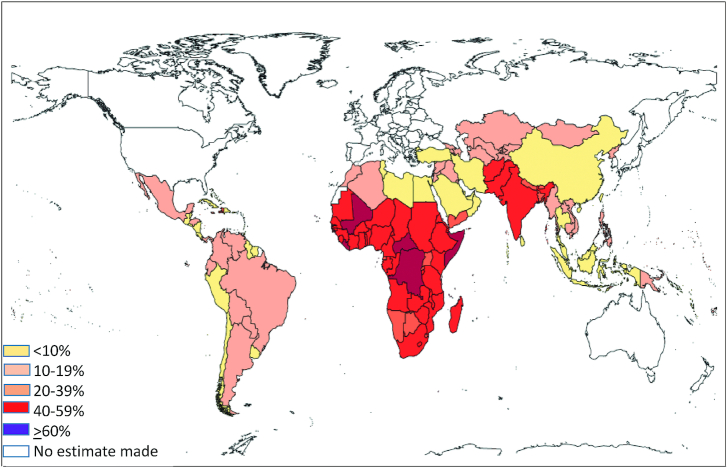
Global prevalence of vitamin A deficiency in children aged 6–59 mo. Reproduced from reference 9 with permission.

In terms of efficacy, programs employing frequent low-dose vitamin A, mimicking food channel delivery, have been most effective in reducing the impact of VAD and elevating serum retinol (20, 39). This evidence raised the option of a potential mix of vitamin A interventions in the areas where VAD is still a public health concern. Regional differences in global trends in VAD, highlighted in a report by Stevens et al. (9), show that whereas the overall prevalence of VAD over the period 1991–2013 was reduced from 39% to 29%, the greatest shifts were occurring in Southeast Asia/Oceania. However, as illustrated in **Figure 4**, the highest prevalence of VAD in 6–59-mo-old children continues to be in South Asia (44%) and sub-Saharan Africa (48%). With specific regard to child mortality rates attributable to VAD, there was an overall decline to 1.7% but virtually all (>95%) of those deaths occurred in Southeast Asia and sub-Saharan Africa (8).

To obtain a clearer picture of global VAD, it is important to see where VAS programs are, and then review their impact on VAD. A recent study by Wirth et al. (6) reported that in two-thirds of the countries currently engaged in VAS programs, either alone or in parallel with 1 other intervention, the data on vitamin A status were either absent or >10 y old. This warrants a call for an update of VAD data for programmatic decision. Table 2 highlights some of the key features of countries currently employing VAS programs.

To guide decisions on country programs, there is a need to address the other critical piece of the program evaluation puzzle—how should vitamin A be assessed by monitoring both prevalence and intervention impact of vitamin A status? Based on a review of the changing vitamin A global landscape, Klemm et al. (66) have drawn several conclusions:

- VAS alone is not a viable sustainable approach to maintain or improve vitamin A status.
- Other interventions are required to be included with VAS to achieve adequacy.
- Decisions on what and how to intervene will depend on:

○ habitual diet surveys that can address such questions as when “lean seasons” in vitamin A security occur, and the timing of peak infectious disease burden;
○ tracking coverage of interventions and including all sources contributing to vitamin A intake;
○ monitoring potential for excess, as well as excessive stores;
○ validation with biochemical indicators with biomarkers that include serum retinol, RBP, breastmilk retinol, retinyl ester, or retinol isotope dilution assay.

- There is a need for vitamin A program assessment guidelines to support decision-making processes at country level with regard to when, what, and how to intervene to improve vitamin A status.

In conclusion, sustainable improvement of vitamin A status is most likely to be attained via enhanced dietary intake through food-based approaches. Given the existing recommendations and recent questions, an overarching question is whether countries should continue to supplement or not, and if not, how would this decision be made by countries? The GAVA Technical Consultation in 2012 recommended that countries should consider scaling back on universal VAS programs when survey data confirm a threshold of verified subclinical VAD prevalence of <5% (based on the WHO VAD public health cutoff for low serum retinol) (58). These questions might be addressed by considering the key data gaps of inadequate up-to-date VAD prevalence, a guide of vitamin A assessment methodologies for both intake (to monitor coverage, adequacy, and excess) and status through the most appropriate biomarkers, and to derive possible algorithms to assist countries in making decisions on the mix of vitamin A interventions (67).

### Country perspectives: implications of the current concern/debate on national efforts to prevent VAD

The experience of countries involved in national programs to address VAD provides a valuable perspective for the issues confronting public health agencies engaged in such efforts. Experiences of 2 countries, Guatemala and Zambia, illustrate the challenges and provide insights about the value of 4 broad elements comprising this enterprise, including: *1*) basic, clinical, and population-based research; *2*) translation of that research into safe and effective interventions; *3*) development, implementation, and evaluation of targeted programs; and *4*) stakeholder support of the roll-out, scale-up, evaluation, and sustainability.

The value of these 2 case studies is that they demonstrate different approaches to the public health problem of VAD—the historically important fortification of sugar from Guatemala (68, 69) and the integrated Zambian approach (70). These examples also demonstrate the value of cross-fertilization of national programs across continents. The sugar fortification component in Zambia was directly influenced by the Guatemalan program, initiated in the late 1960s, sharing expertise to launch the Zambia fortification of sugar with vitamin A, which has been mandatory in Zambia since 1998 (largely supported by the United States Agency for International Aid) (71). Both experiences demonstrated the implications of the current concerns and resulting debate on national efforts to efficiently prevent VAD in their countries.

During the 1960s, VAD was recognized as a public health problem in Guatemala and other Central American countries, as a consequence of a monotonous cereal-based diet poor in both animal and vegetable sources of vitamin A. Results of national nutrition surveys carried out in the mid-1960s in Central America showed 42–69% of families with vitamin A consumption <25% adequacy, severely affecting children aged 0–9 y, particularly in rural areas (72). In Guatemala, white sugar was identified as a food vehicle largely consumed by most of the population (including most at-risk groups), and although it had never before been fortified with vitamin A, the groundwork leading to its fortification was successfully undertaken by scientists at the Institute of Nutrition of Central America and Panama, and the national fortification program was started in 1965 (69). A longitudinal evaluation demonstrated the effectiveness of the strategy (73), and follow-up biochemical assessments based on national health and micronutrient surveys showed a decline of VAD from 26.0% to 15.8% in children aged 0–59 mo during the following 30-y period (1965–1995). The level of vitamin A fortification in sugar has remained the same since the program was initially launched (15 mg/kg at the production site), whereas sugar consumption has increased from 20–40 g/d in 1976 to ∼100 g/d in 2005. Since the early 2000s, Guatemala also has had a national policy to provide VAS to children aged 6–59 mo, linked to their immunization schedule. VAS coverage varies by year and age group: between 2011 and 2015, children aged 6–12 mo had coverage of 50–74%, whereas for children aged 1–2 y it was 27–40%, and for children aged 2–5 y coverage was in the range of 14–21%. In response to these situations, the Ministry of Health now supplements only children aged 6–24 mo, and the level of fortificant added to sugar is being revised (74, 75). In 2010, the prevalence of low serum retinol values in children aged 6–59 mo was 0.3% (76), and the country is now considered free of VAD. Having monitoring and surveillance systems in place has been an important component in the sustainability and success of the program (72).

In Zambia, VAD has been addressed as a problem of public health significance, leading to different approaches that include vitamin A capsule supplementation targeting since 1990 children aged 6–59 mo, mandatory fortification of sugar with vitamin A targeting the general population since 1998, and biofortification of maize and orange-fleshed sweet potato with provitamin A carotenoids for the general population in selected areas of the country since 2007 (14, 63). To support these interventions, the country has laid emphasis on nutrition education programs encouraging the population to demand vitamin A–related services and adopting behavior change after public communication around diet diversification with a focus on promotion of foods rich in vitamin A (71). Overlap of different strategies has led to high vitamin A status in some areas and population groups (49). Furthermore, the latest national nutrition survey was conducted in 2003, so there are no recent data to document vitamin A status in the population.

As illustrated, both countries have used various strategies to address VAD. For example, both have implemented high-dose supplementation programs for children and even, more recently, micronutrient powder provision, on top of the ongoing large-scale food fortification, but with variable coverage of the various approaches. This has led to some inefficiencies, as well as concerns about overdosing and even toxicity, especially as more commercial foods have been increasingly fortified voluntarily (77). The debates on these questions have several implications for both research and policy. The experiences in both countries highlight the importance of ongoing monitoring activities that provide information on the quality and coverage of the program and epidemiological surveillance. Guatemala has been examining in detail actual coverage levels of supplementation and fortification activities, confirming that universal sugar fortification with vitamin A has been a sustainable cost-effective strategy in drastically reducing VAD and has overcome several attempts to interrupt the program (69, 78–80). However, Guatemala is currently more concerned with the possibility of excess vitamin A intakes as the availability increases from various sources, and is looking at adjusting the amount of fortificant (and so reducing the needed intake of the vehicle) in the light of sugar's role in NCDs and emerging obesity, especially in women of reproductive age in the country. At the same time, in Zambia there is currently no consensus on doing this, because questions remain on how to assess the contribution of VAS to morbidity and mortality reduction among children, how to control risks of excess intakes, how to track the delivery of interventions such as the availability of vitamin A from the successful biofortification in Zambia (81, 82), and how best to design and implement an integrated monitoring and evaluation system (63, 83). As in other countries, concern has been raised in Zambia as to whether to continue with vitamin A capsule supplementation among children aged <5 y in light of questions about the impact of this intervention on morbidity and mortality among children. Both countries’ programs demonstrate valuable lessons and the need for continuing monitoring and modification of programs for sustainability and effectiveness, as well as for providing information to guide public policies. Published reviews have highlighted that coverage, utilization, and compliance are all neglected areas of evaluation in fortification programs (12, 84).

### Context and perspectives on program implementation

As noted, there are vitamin A programs in about ∼80 countries but there are significant differences in the implementation, coverage, and impact (19). Given the alternative, but often complementary, possible interventions for helping address VAD, when new research, new epidemiological data, or other drivers, such as funding, dictate a change in public health intervention, care must be taken to ensure that the replacement interventions represent an improvement (i.e., an ineffective program should not be replaced with another of lower performance). The goal should remain to provide vitamin A adequacy through diverse diets including fortified foods. Somewhat similar to the experience in Guatemala and Zambia above, Madagascar was faced with a need to explore other vitamin A intervention options (85). The intervention, micronutrient powders in a marketplace-based approach, was problematic due to product batches of poor quality causing issues around acceptability. However, the presence of the existing program has fostered an improved understanding of micronutrient nutrition, including vitamin A, in the home that appears to have had a secondary impact of improving dietary diversity and presumably child nutritional status.

**FIGURE 5:**
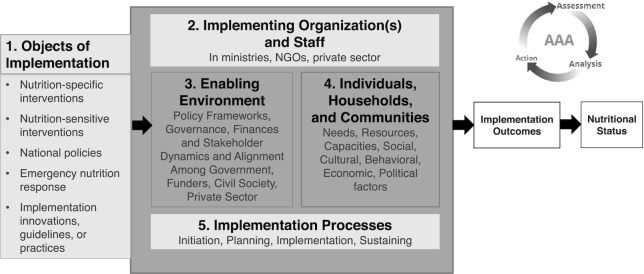
The 5 domains whose characteristics, capacities, dynamics, and fit affect implementation quality. NGO, nongovernmental organization. Adapted from reference 88 with permission.

It is also the case that interventions that are extremely successful in some settings might not be in others. For example, reports from a study in Kenya (86, 87) describe the factors contributing to a dramatic drop in uptake of weekly micronutrient sachets (containing vitamin A) from nearly 100% to 30% (stabilizing between 45% and 52% with increased social marketing efforts). The intervention was delivered at a refugee camp and was unsuccessful in terms of addressing VAD (as well as other key nutrients targeted). Among the factors contributing to this failure of an intervention that had been successful in other settings was the lack of consideration of the context, in this case the challenging setting of a refugee camp. These and other experiences call for strengthened implementation science in nutrition using a wide range of methods to identify and address implementation bottlenecks (88), which can help successful program scale-up, as illustrated in **Figure 5**.

Linking effective coverage estimates with an adapted Lives Saved Tool (LiST) method has been found to permit estimation of the effects of combinations of vitamin A programs (beyond VAS programs only) on child mortality to aid program planning and management (23). Serious program monitoring and evaluation are necessary to confirm predicted outcomes. Key elements that need to be adhered to by the implementing community for success should include:

- Vitamin A status assessment using tools that are less affected by inflammation, for example, MRDR, isotope dilution.
- Surveillance assessing vitamin A exposure including: total intake, coverage of VAS, type of intervention [e.g., micronutrient powders, lipid nutrition supplements, (bio)fortified foods], and, when appropriate and available, issues such as the form, content, and bioavailability estimates.
- Innovative survey designs and point-of-care status assessment to modify existing programs:

○ subnational data at community or district level;
○ confirming that where VAD prevalence is rare, VAS should be scaled back, not least for cost-effectiveness of programs;
○ where VAS exits, other targeted interventions can be added but need to be closely monitored.

- Increased use and application of existing and emerging tools:

○ Fortification Assessment Coverage Toolkit (89);
○ modeling tools to optimize national micronutrient intervention strategies such as using the LiST for micronutrients (23).

## Discussion

This article can be summarized broadly as demonstrating a clear need for more adequate and improved prevalence data at the country level to inform and strengthen program/policy and interventions. Clearly, improved and contemporary data are also essential when countries are planning when and how best to transition from one type of program to another combination of interventions. At a global level, reliable, comparable data allow an updated view of vitamin A status around the world, and technical agencies such as the WHO will lead in taking the steps to help ensure this need is met. The recent global report from UNICEF (19) showed, amongst other disturbing figures, that only 64% of children in need (as defined in the WHO e-Library of Evidence for Nutrition Actions guidance) (90) are actually receiving vitamin A supplementation, which means >140 million children who are likely vitamin A deficient are missing out. An improved capacity to strengthen monitoring to identify these unreached children would also contribute to building knowledge on the most effective platforms for reaching at-risk children aged 6–59 mo.

Among other challenges, concern exists about the responsiveness, or lack thereof, of serum retinol as a biomarker, and the lack of reliability of RBP as a surrogate biomarker of vitamin A status (37, 60). More information is needed to document if MRDR with a cutoff, which seems to be a more sensitive indicator of response to interventions, is sufficient for monitoring. Although there are many different kits for RBP available, limitations continue to exist with regard to standardization. It has been reported that in southern Thailand, MRDR has been used to monitor response to a targeted supplementation program (E Udomkesmalee, unpublished results, 2016). The results of analysis of surveillance indicated a population response and reinforced the value of a combination of assessment methods to determine need and response to avoid unintended consequences and to support decisions about what, when, and how to intervene. It is important to reinforce the notion that it is possible to deploy multiple biomarkers, for example, serum retinol, RBP, and MRDR, as well as biomarkers of inflammation to assess vitamin A in the field.

Early studies of vitamin A interventions assessed all-cause mortality and provided evidence that these interventions were effective in preventing child deaths from some infectious diseases, namely, acute diarrhea and measles. The current reputed lack of response in all-cause mortality (32) might be because the most significant effects are limited to severe forms of infection. As these studies suggest, the lack of response might be associated with a changing global health landscape, with reductions in prevalence of these severe infections, especially measles (91). Although this then begs the question, “How much is VAS actually contributing to that specific aspect of the global health landscape?” in countries with continued high rates of mortality, there should be a “high bar” for making decisions to change VAS programs.

In addition to the concern about efficacy are concerns about the safety of VAS, particularly in specific vulnerable groups, for example, infants and young children (92, 93). Concerns have also been raised about multiple sources of vitamin A increasingly available, and the previous practice of “piggybacking” VAS onto child immunization programs, because it was proposed that VAS had a potentially harmful effect when provided with inactivated vaccines (such as DTP), whereas it provided beneficial effects when combined with live vaccines (such as measles) (92, 94), but evidence suggests that these do not seem to be significant issues (93). The use of multiple programs does, however, remain a partly unresolved issue (95), which emphasizes the importance of monitoring and the need for both effective surveillance and a targeted research agenda. This is perhaps especially so because, based on the emerging science, governments, implementation, and nongovernmental agencies are being encouraged to move away from siloed, single nutrition-focused interventions to deployment of more integrated approaches using nutrition-specific and nutrition-sensitive programs.

## Conclusions

The clinical impact and public health implications of VAD present a complicated set of challenges for biology, assessment, and public health surveillance and policy. The reliance on biomarkers of questionable utility, and the use of the resulting data to make decisions about standards of care and public health interventions are potentially problematic in terms of both safety and efficacy. Moreover, using sentinel, albeit nonspecific, indicators such as mortality—because they can be affected by a myriad of nonnutrition factors—is problematic, particularly in LMICs. Among the many factors contributing to poor vitamin A status are the negative impact of infectious diseases on appetite, increased excretion, and increased metabolism (20). The effects that VAS can have on modulating the immune system have been summarized (4). Of critical importance in this context is that these effects can be sensitive to the amount and timing of the VAS dose, and can be influenced by sex and proximity to other interventions such as live (e.g., measles vaccine) or nonlive vaccines (like the DTP vaccine) (27).

With specific reference to nutritional assessment, the increasing appreciation of the need to account for the presence of inflammation and its impact on interpretation of biomarkers of vitamin A status will need to be incorporated into assessments at clinical and population levels (61).

Although dietary insufficiency of vitamin A is more likely in areas of general endemic food/nutrition insecurity, it must also be recognized that the health context of these settings is becoming increasingly complex with often unabated prevalence of infectious diseases and increasing prevalence of NCDs, all colliding at individual and population levels. This complexity, increasingly exacerbated by climate change, demands vigilance in terms of both assessment and interventions.

Although there will likely need to be a scaling-up of interventions to complement VAS programs, depending on continuing or emerging needs, poor access of disadvantaged populations will also affect the likely availability of supplements—food supplements such as micronutrient powders and lipid-based therapeutic and supplementary foods—as well as vitamin A–fortified oils and foods. Social and cultural factors and low-capacity health and public health nutrition systems add yet another group of factors that have perhaps not been adequately factored in the design of past programs.

The debate over whether it is time to consider transitioning from supplementation to other intervention options in some national settings is timely. Concerns have been raised about availability of vitamin A capsules in many countries. UNICEF recently reported a declining coverage in certain settings. The knowledge that more physiological doses through diet and fortification will offer sustainable adequate vitamin A status suggests that, at the very least, nutrition education about the most appropriate and sustainable sources of vitamin A will need to be context specific, reflecting the health and nutrition status of the target groups. Dietary approaches will be contingent upon a sufficient and varied food supply, including liver, eggs, dairy products, green leafy vegetables, and fruits, as well as fortification and biofortification (depending on the national situation).

Both nutrition-specific and nutrition-sensitive interventions such as horticultural approaches including homestead gardening, need to be promoted in complementarity with one another. Social and cultural approaches to reduce gender inequity and strengthen both general and specific education will be critical. The impact of climate change is likely to be negative both in terms of food systems and health, and will require a proactive response (96). An important first step is to update current knowledge of the vitamin A status in countries, and perhaps especially those with supplementation programs. At the very least, the I-to-I approach encourages identification of the many factors involved and promotes a multisectoral approach. Ultimately, improved equity will be the most sustainable, if challenging, underpinning to improved health and nutrition.

The implementation of the I-to-I approach recognizes the need for, and value of, input from numerous sources of data that represent the efforts of each of the key stakeholder communities. Conceptually this approach makes sense. In so doing, we stand a better chance of not only assessing safety and efficacy in the moment, but also avoiding unintended consequences in the future.

## Notes

Supported by the Bill & Melinda Gates Foundation.

Author disclosures: DRR, ID-H, SAT, PSS, EU, CM, DIM, MM, KK, and HM, no conflicts of interest.

Perspective articles allow authors to take a position on a topic of current major importance or controversy in the field of nutrition. As such, these articles could include statements based on author opinions or point of view. Opinions expressed in Perspective articles are those of the author and are not attributable to the funder(s) or the sponsor(s) or the publisher, Editor, or Editorial Board of *Advances in Nutrition*. Individuals with different positions on the topic of a Perspective are invited to submit their comments in the form of a Perspectives article or in a Letter to the Editor.

Abbreviations used: BRINDA, Biomarkers Reflecting Inflammation and Nutritional Determinants of Anemia; DTP, diphtheria-tetanus-pertussis; GAVA, Global Alliance for Vitamin A; I-to-I, integration to effective implementation; LiST, Lives Saved Tool; LMIC, low- and middle-income country; MRDR, modified relative dose response; NCD, noncommunicable disease; RBP, retinol-binding protein; RUTF, ready-to-eat therapeutic food; VAD, vitamin A deficiency; VAS, vitamin A supplementation.
